# Radiotherapy among nonagenarians with anal or rectal carcinoma: should we avoid or adapt treatment?

**DOI:** 10.1186/s40880-017-0224-5

**Published:** 2017-07-14

**Authors:** Alexis Vallard, Chloé Rancoule, Jean-Baptiste Guy, Avi Assouline, Alexander T. Falk, Pierre Auberdiac, Julien Langrand-Escure, Cyrus Chargari, Nicolas Magné

**Affiliations:** 1 Department of Radiotherapy, Lucien Neuwirth Cancer Institute, 108 Bis, Avenue Albert-Raimond, BP 60008, 42271 Saint-Priest En Jarez, France; 2 Department of Radiotherapy, Porte De Saint Cloud Clinical Centre, 92100 Boulogne-Billancourt, France; 3 Department of Radiation Oncology, Antoine Lacassagne Centre, 06100 Nice, France; 4 Department of Radiotherapy, Claude Bernard Private Hospital, 81000 Albi, France; 5 Department of Radiotherapy, Val-De-Grâce Military Hospital, 75230 Paris, France

**Dear editor,**

Radiotherapy is a cornerstone in the management of anal or rectal cancer. Because elderly patients are often excluded from clinical trials, little is known about radiotherapy’s therapeutic index (efficacy/toxicity ratio) in the geriatric population [1]. Still, the ageing of population imposes the challenge to treat older cancer patients and probably to adjust their treatment [2]. A few studies reported data on radiation-induced toxicities in nonagenarian patients, but data on efficacy are still scarce. For rectal cancer, preoperative radiotherapy programs were suggested to be feasible in elderly patients [3–5], with a preference for short protocols without chemotherapy [4]. For anal cancer, the safety of radiochemotherapy was only reported in limited sets of elderly patients [6]. The objective of the present study was to report efficacy and toxicity data on the radiotherapy treatment of nonagenarian patients with anal or rectal cancer.

We analyzed the data of 34 nonagenarian patients with anal or rectal cancers: 27 (79.4%) with a rectal adenocarcinoma and 7 (20.6%) with an anal canal squamous cell carcinoma. At the time of irradiation treatment, the patients’ mean age was 92.7 years (standard deviation, 2.3 years). Before radiotherapy, 19 patients (55.9%) had an Eastern Cooperative Oncology Group Performance Status score of two or higher. Eleven patients (32.4%) were nursing home residents. Primarily, 16 patients (47.1%) were diagnosed with a locally advanced tumor (T3-4 or N1-3) and 6 (17.6%) with a metastatic disease. Seven patients (20.6%) underwent surgery before radiotherapy. Patient characteristics are shown in Table 1.

**Table 1:** Characteristics of 34 nonagenarians undergoing radiotherapy for anal or rectal carcinoma

Patient characteristic	No. of patients (%)
Gender	
Male	16 (47.1)
Female	18 (52.9)
Performance status score	
0–1	14 (41.1)
2–4	19 (55.9)
Not reported	1 (3.0)
Living place	
Home	22 (64.7)
Nursing home	11 (32.3)
Not reported	1 (3.0)
Tumor characteristic	
Primary site	
Rectum	27 (79.4)
Anal canal	7 (20.6)
Stage	
Localized (T1-2N0)	7 (20.6)
Locally advanced (T3-4 or N1-2)	16 (47.1)
Metastatic	6 (17.6)
Not reported	5 (14.7)

Three-dimensional conformal radiotherapy was used for curative (*n* = 13, 38.2%) and palliative intents (*n* = 21, 61.8%). The median delivered dose was 43.5 Gy (range 6.0–64.0 Gy), and the median biologically equivalent dose in 2.0 Gy fractions (EQD2) was 44.7 Gy_α/β=10_ (range 8.0–64.0 Gy_α/β=10_). The median number of fractions was 14 (range 1–32), and the median dose was 3.0 Gy per fraction (range 1.8–10.0 Gy per fraction), with 13 patients (38.2%) receiving a dose less than 2.5 Gy per fraction. Median total treatment duration was 3.0 weeks (range 0.1–6.6 weeks). No concomitant chemotherapy was administered.

In the entire cohort of 34 patients, 3 (8.8%) discontinued treatment: 2 because of patients’ noncompliance and 1 caused by an acute grade 3 toxicity. Eleven patients (32.3%) had a follow-up exceeding 6 months and were evaluated for late toxicity: 1 (2.9%) developed a grade 3–4 late fecal incontinence, 4 (11.8%) developed a grade 1–2 late toxicity (pelvic fibrosis, urinary incontinence, and fecal incontinence), and 6 (17.6%) did not report any late complication.

Median follow-up time was 13.4 weeks (range 0–142.0 weeks), with a follow-up less than 4.0 weeks for 8 patients (23.5%). At the last follow-up, tumor control (defined as stable disease, partial response, and/or complete response) was achieved for 18 patients (52.9%), including 10 of the 13 patients treated in curative intent and 8 of the 21 patients treated with palliative intent; tumor-related symptoms were controlled in 13 patients (61.9%) who underwent a palliative radiotherapy. Nine patients (26.5%) had died at the last follow-up; of them, 8 (88.9%) had disease progression.

In this study, we retrospectively assessed the safety and efficacy of radiotherapy for 34 nonagenarian patients with anal or rectal cancer. We observed only infrequent infield late toxicities (0% grade 5, 2.9% grade 3–4, and 11.8% grade 1–2). At the last follow-up, disease was controlled in 52.9% of the 34 patients. As expected, hypofractionated programs were widely used, since they reduce acute toxicities (in cell populations with a high turnover, such as mucosal membranes) and favor radiotherapy completion. Our results suggest that radiotherapy is feasible in nonagenarian patients with anal or rectal cancer, but geriatric assessment could probably decrease the probability of discontinuing treatment. Although no consensus exists regarding the role of brachytherapy in the management of anal cancer in patients 90 years of age or older, it should probably be considered a major option after external beam radiotherapy for patients with good physical condition, since very limited toxicities and good results on efficacy were suggested by recent studies [7, 8].

In conclusion, based on these real-life findings, radiotherapy may be feasible in nonagenarians patients, either with cure or palliative intent.
